# Radiomic study of common sellar region lesions differentiation in magnetic resonance imaging based on multi-classification machine learning model

**DOI:** 10.1186/s12880-025-01690-5

**Published:** 2025-05-03

**Authors:** Hang Qu, Qiqi Ban, LiangXue Zhou, HaiHan Duan, Wei Wang, AiJun Peng

**Affiliations:** 1 Department of Radiology, Affiliated Hospital of Yangzhou University, Yangzhou University, Yangzhou, Jiangsu province 225000 China; 2 Department of Neurosurgery, West China Hospital, Sichuan University, Chengdu, Sichuan province 610065 China; 3 College of Computer Science, Sichuan University, Chengdu, Sichuan province 610065 China; 4 Department of Neurosurgery, Affiliated Hospital of Yangzhou University, Yangzhou University, No.368, Hanjiang Middle Road, Hanjiang district, Yangzhou city, Jiangsu province 225000 China

**Keywords:** Common sellar region lesions, Radiomics, Extreme gradient boosting

## Abstract

**Objective:**

Pituitary adenomas (PAs), craniopharyngiomas (CRs), Rathke’s cleft cysts (RCCs), and tuberculum sellar meningiomas (TSMs) are common sellar region lesions with similar imaging characteristics, making differential diagnosis challenging. This study aims to develop and evaluate machine learning models using MRI-based radiomics features to differentiate these lesions.

**Methods:**

Two hundred and fifty-eight pathologically diagnosed sellar region lesions, including 54 TSMs, 81 CRs, 61 RCCs and 63 PAs, were retrospectively studied. All patients underwent conventional MR examinations. Feature extraction and data normalization and balance were performed. Extreme gradient boosting (XGBoost), support vector machine (SVM), and logistic regression (LR) models were trained with the radiomics features. Five-fold cross-validation was used to evaluate model performance.

**Results:**

The XGBoost model showed better performance than the SVM and LR models built from contrast-enhanced T1-weighted MRI features (balanced accuracy 0.83, 0.77, 0.75; AUC 0.956, 0.938, 0.929, respectively). Additionally, these models demonstrated significant differences in sensitivity (*P* = 0.032) and specificity (*P* = 0.045). The performance of the XGBoost model was superior to that of the SVM and LR models in differentiating sellar region lesions by using contrast-enhanced T1-weighted MRI features.

**Conclusion:**

The proposed model has the potential to improve the diagnostic accuracy in differentiating sellar region lesions.

**Supplementary Information:**

The online version contains supplementary material available at 10.1186/s12880-025-01690-5.

## Introduction

The most common sellar region lesions, including pituitary adenomas (PAs), craniopharyngiomas (CRs), Rathke’s cleft cysts (RCCs) and tuberculum sellar meningiomas (TSMs), may present with similar clinical symptoms [1, 2]. Although physical and laboratory examinations are important in the diagnosis of these diseases, magnetic resonance imaging (MRI) is a particularly vital tool for obtaining a precise diagnosis.

Some PAs can infiltrate the sellar floor, cavernous sinus, and suprasellar region, resembling TSMs. Additionally, approximately 48% of PAs contain cystic components [3], and due to acute or chronic bleeding, they may exhibit varying signal intensities on T1- or T2-weighted MRI. CRs and RCCs also show diverse signal characteristics on MRI due to differences in lesion composition and intracystic protein levels [4]. These similarities in imaging features pose challenges for differential diagnosis [5]. Wen et al. [6] reported that 50% of RCCs were preoperatively misdiagnosed as PAs and 13.6% as CRs. Accurate diagnosis of these lesions is crucial as the surgical approach or treatment varies depending on the specific disease.

Previous studies have demonstrated that machine learning (ML) performs well in classifying and predicting PA subtypes on T2-weighted images (T1WI) [6, 7]. Huang et al. [4] showed good performance in diagnosing CR pathological subtypes on T1-weighted images (T1WI). However, there has been only one study [8] utilizing ML to analyze anterior skull base lesions based on contrast-enhanced T1-weighted images (CE-T1WI), and most prior studies relied on only a single MRI sequence.

Radiomics can be used to extract high-dimensional features from MRI of different sequences, which may help to improve diagnostic accuracy. This study is to develop ML models that can differentiate common sellar region lesions, including PAs, CRs, RCCs, and TSMs, using radiomics features from MRI. We hypothesize that ML, particularly with CE-T1WI, can improve diagnostic accuracy and assist in clinical decision-making by reliably distinguishing between these lesions, ultimately enhancing patient outcomes.

## Materials and methods

### Patient population

A series of 259 patients with preoperative MR images and common sellar lesions confirmed by postoperative pathology were enrolled from the Neurosurgery Department of the West China Hospital, Sichuan University, between January 2016 and February 2021. The lesions included 54 cases of TSM, 81 cases of CR, 61 cases of RCC and 63 cases of PA.

The inclusion criteria were as follows: (1) diagnosis confirmed by postoperative pathology; (2) MR images of sufficient quality to provide lesion information; and (3) all MR images obtained within one week before surgery. The exclusion criteria were as follows: (1) previous operations or radiosurgery, (2) lesion diameter less than 1 cm [9], and (3) obvious artifacts on MR images.

### Clinical MRI assessment

All patients had undergone MRI examinations, including T1WI, T2WI, and CE-T1WI, on a device (Siemens Trio, 3.0 T, Germany) and a cranial MRI coil. All images were obtained with a 2D spin-echo sequence in coronal MRI mode. The parameter settings for each sequence were as follows: (1) T1WI: repetition time (TR) 600 ms, echo time (TE) 8.1 ms, field of view (Fov) 200 mm, Voxel size 0.8*0.6*2.0 mm; (2) T2WI: TR 4000 ms, TE 93 ms, Fov 220 mm, Voxel size 0.8*0.6*2.0 mm; (3) CE-T1WI: TR 232 ms, TE 8.1 ms, Fov 200 mm, Voxel size 0.9*0.6*2.0 mm.

### Lesion delineation and radiomic feature selection

ITK-SNAP software (version 3.8.0, www.itk-snap.org) was used to load all MRI sequences. The sellar region lesions of each slice on each MRI sequence were delineated as the region of interest [10]. The delineation of the ROI was performed by comparing different sequences and carefully separating the lesion from adjacent brain tissues using surrounding anatomical structures as references.

One neurosurgeon (with 14 years of working experience) and one neuroradiologist (with 13 years of working experience) performed this manual delineation. Then, another expert neurosurgeon and radiologist reviewed the results together. Any disagreements regarding the lesion boundaries were documented and resolved by the senior neurosurgeon and radiologist.

The extraction of radiomic features was based on the segmentation results described in the previous paragraph. Using the Simple ITK software library (http://www.simpleitk.org/), individual DICOM images of each MRI sequence for each patient were loaded and integrated into a three-dimensional near-raw raster data (NRRD) image. Similarly, each image slice with an ROI mask was processed to generate a three-dimensional labeled NRRD image. All three sequences were acquired using the same localization images during scanning, facilitating uniform ROI delineation across the sequences. Subsequently, the images were standardized and subjected to wavelet transformation.

MRI images from 40 randomly selected patients (10 cases each of TSM, CR, RCC, and PA) were used to assess intra- and inter-observer consistency. ROIs on T1WI, T2WI, and CE-T1WI were independently segmented by a neurosurgeon and a neuroradiologist within the same time frame to evaluate inter-observer agreement for radiomic feature extraction. To assess intra-observer reproducibility, the neurosurgeon re-delineated the ROIs following the same protocol after a two-week interval. Agreement was evaluated using the intraclass correlation coefficient [11], with features achieving an ICC > 0.75 considered to have good reliability [12]. Upon analysis, all extracted features demonstrated ICCs above 0.75.

PyRadiomics 1.2.0 (https://pyradiomics.readthedocs.io/) was used to extract radiomics features from the images in each MR sequence. After feature extraction, a total of 100 features were obtained from the original images of each MRI sequence, which are shown in Table 1. In addition, 688 texture features of the same type were extracted from 8 wavelet-transformed images (688/8 = 86 features per transformation, which did not include shape features). Therefore, 788 individual radiomic features were extracted from each MRI sequence.

**Table 1:** Radiomics features in MRI images

Radiomics features	Quantity
First-order statistical features	18
Shape features	14
Gray level cooccurrence matrix features	22
Gray level run length matrix	16
Gray level size zone matrix	16
Gray level dependence matrix	14

### Data processing

All patients were randomly divided into 5 subsets, of which 4 were randomly used for training the model, while the remaining subset was used for validation. First, the training set was normalized with standard software (https://scikit-learn.org/stable/modules/preprocessing.html). To balance the data, number of the TSM, RCC, and PA samples in the training set was increased to 65 through the SMOTE [13] algorithm (https://pypi.org/project/imbalanced-learn//), the number of samples in the CR training set. Subsequently, after training, the normalized model was applied to the validation set.

### Machine learning methods and model development

Three machine learning methods were used for model development based on their proven ability to deliver high and stable performance in medical imaging studies [14]. (1) Support vector machine, SVM (https://scikit-learn.org/stable/modules/svm.html, scikit-learn software packages), (2) Logistic regression, LR (https://scikit-learn.org/stable/modules/linear_model.html#logistic-regression), (3) Extreme Gradient Boosting, XGBoost, (https://xgboost.readthedocs.io/en/latest/). The parameter settings for XGBoost included the gbtree tree model as the base classifier, n_estimatores = 400, max_depth = 10, learning_rate = 0.2, and the remaining parameters were set to default values.

**Fig. 1:**
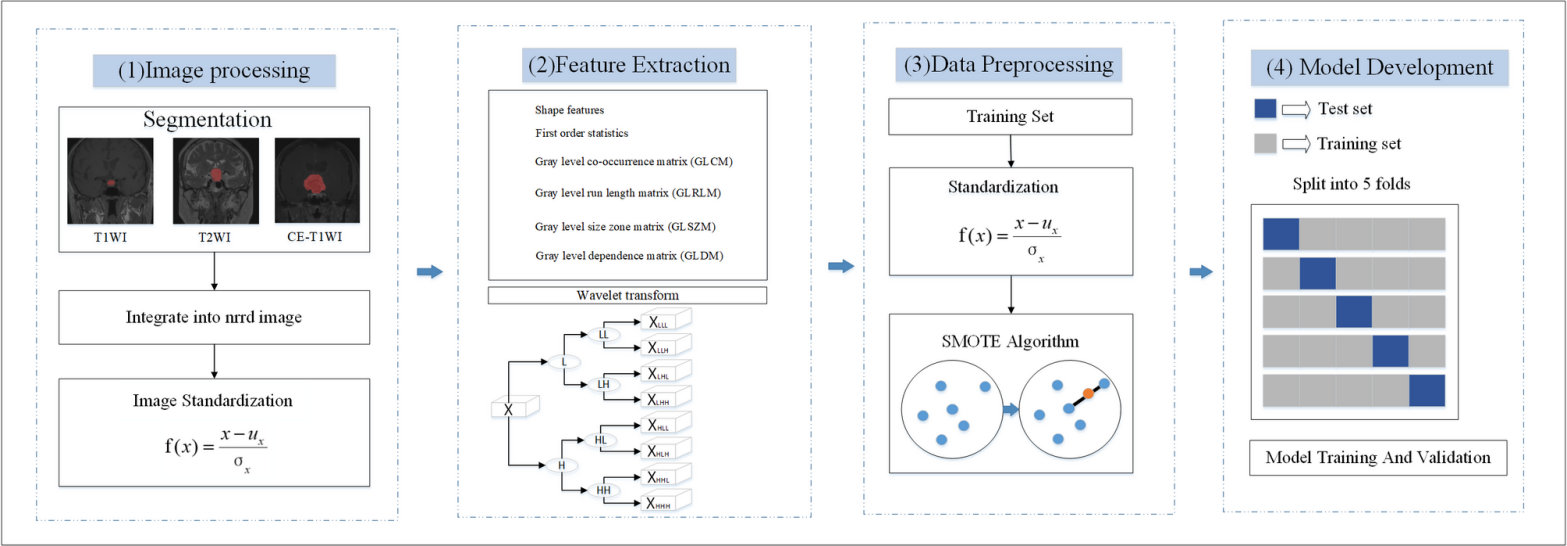
The overall process of radiomics processing

Five-fold cross-validation was used for all models to evaluate their performance in the differential diagnosis of sellar lesions. In our study, the training and testing datasets in each fold of the five-fold cross-validation were strictly independent, with no overlap of patient data between the two, ensuring an unbiased evaluation of model performance. The overall flow of the radiomics processing is shown in Fig. 1.

To assess computational efficiency, inference time was measured on the test subset of the best-performing fold during the five-fold cross-validation. GridSearchCV was used to optimize model hyperparameters within each training set of the folds [15]. After the optimal configuration was identified, the final model from the best fold was applied to its held-out test data, and the forward inference time was recorded. Since the variation in inference time across different folds was minimal, the reported value provides a representative estimate of the model’s runtime performance in real-world applications.

### Hardware and software setup

All computations were performed on a desktop server equipped with an NVIDIA GTX 1080Ti GPU (11 GB GDDR5X, 64 GB RAM, Ubuntu 18.04). The implementation of the model was carried out in Python 3.7, using Keras (https://keras.io/)and Tensorflow (https://www.tensorflow.org/) open-source libraries.

### Statistical methods

Continuous variables are expressed as the means ± standard deviations with SPSS v.23.0 software (Armonk, New York, United States). The nonparametric Kruskal‒Wallis H test was used to evaluate the sensitivity, specificity, and accuracy, and a two-sided P value < 0.05 was considered to indicate statistical significance. Balanced accuracy normalizes the true positive rate and the true negative rate by the number of positive and negative samples and divides the sum into two parts.

$${\rm{Balanced}}\,{\rm{accuracy = }}\>{{{\rm{TPR}} + {\rm{TNR}}} \over 2}$$

$${\rm{TPR:}}\,{\rm{ True}}\,{\rm{ positive}}\,{\rm{ rate, }}\,{\rm{TNR: }}\,{\rm{True}}\,{\rm{ negative}}\,{\rm{ rate}}$$

A confusion matrix was created to evaluate the performance in differentiating sellar lesions for each MRI sequence, including sensitivity, specificity, and accuracy. Additionally, the area under the receiver operating characteristic (ROC) curve (AUC) was calculated. The macroaveraged ROC curve was used to evaluate the performance of the multiclass classifier. To statistically validate the AUC values, we adopted the nonparametric method proposed by Hanley & McNeil [16], and a macro-averaged AUC with its 95% confidence interval was calculated based on the t-distribution [17].

## Results

### Clinical characteristics of the four sellar lesions

The clinical characteristics of patients with sellar lesions are shown in Table 2. Among female patients, the incidence of TSMs (57.41%), RCCs (55.74%) and PAs (53.97%) was slightly higher than that of CRs (48.15%), but the difference was not significant (*P* = 0.745). The average age of the patients with CR was 51.62 years, which was younger than that of patients with the other three sellar lesions. The average diameter of the RCCs was 2.48 cm, which was the largest among the sellar lesions. However, the differences among the diameters of these four lesions were not significant (*P* = 0.754). The clinical laboratory examination data is shown in the supplementary file (Table 1).

**Table 2:** Clinical characteristics of patients with four sellar lesions

Variables	TSMs	CRs	RCCs	PAs	*P*-Value
Sex(%)					0.745
Male	23(42.59%)	42(51.85%)	27(44.26%)	29(46.03%)	
Female	31(57.41%)	39(48.15%)	34(55.74%)	34(53.97%)	
Age	46.67 ± 11.59	51.62 ± 18.55	43.64 ± 14.77	45.98 ± 13.52	0.509
Diameters (cm)	2.16 ± 0.67	2.09 ± 0.64	2.48 ± 0.77	2.06 ± 0.70	0.754

### Balanced accuracy and confusion matrix for each ML model

**Fig. 2:**
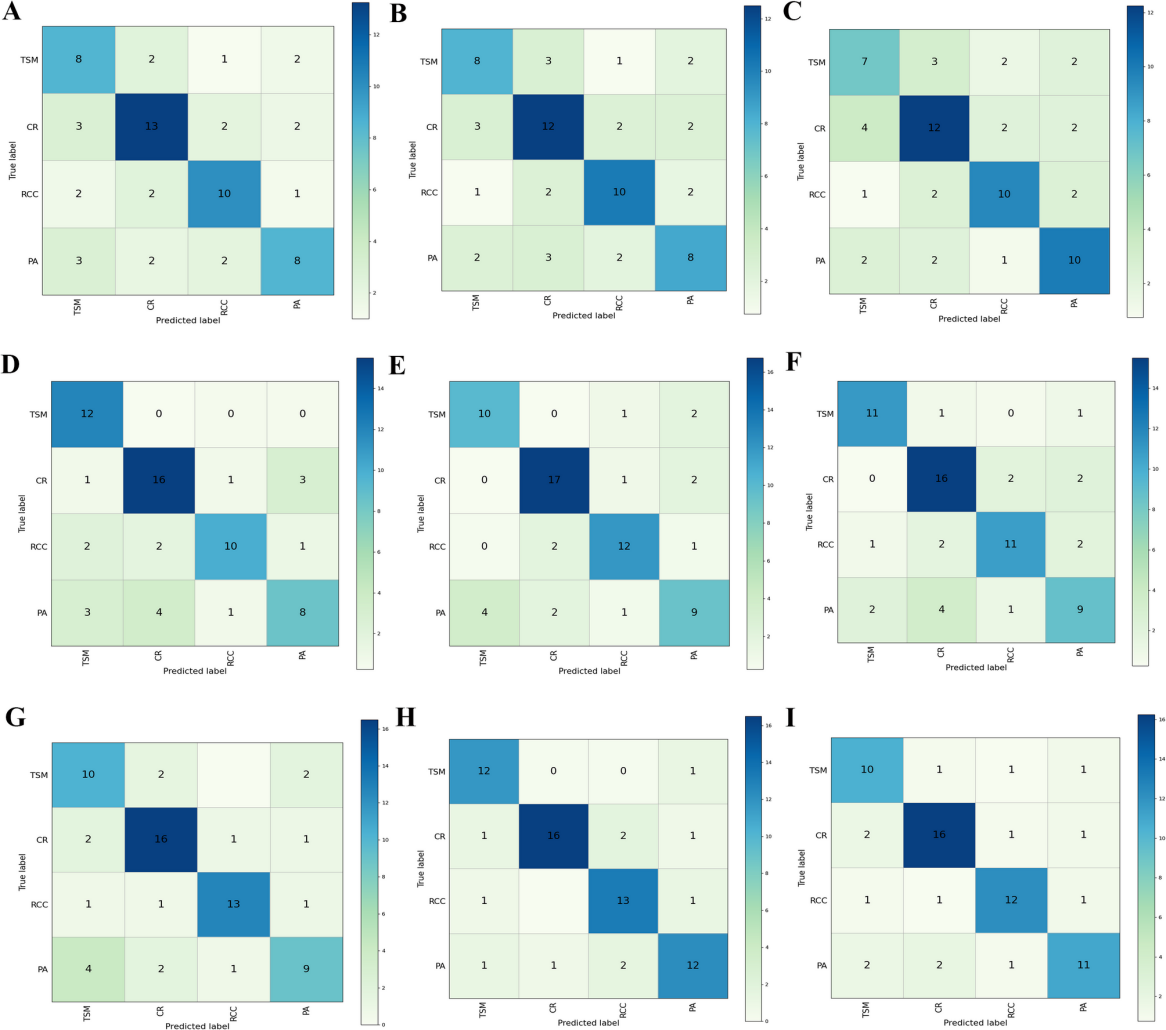
Mean confusion matrix for the three models in different MRI sequences. Mean confusion matrix in SVM model (**A, D** and **G**), in XGBoost model (**B, E** and **H**) and in LR model (**C, F** and **I**) in T1, T2-weighted and contrast-enhanced T1-weighted

**Fig. 3:**
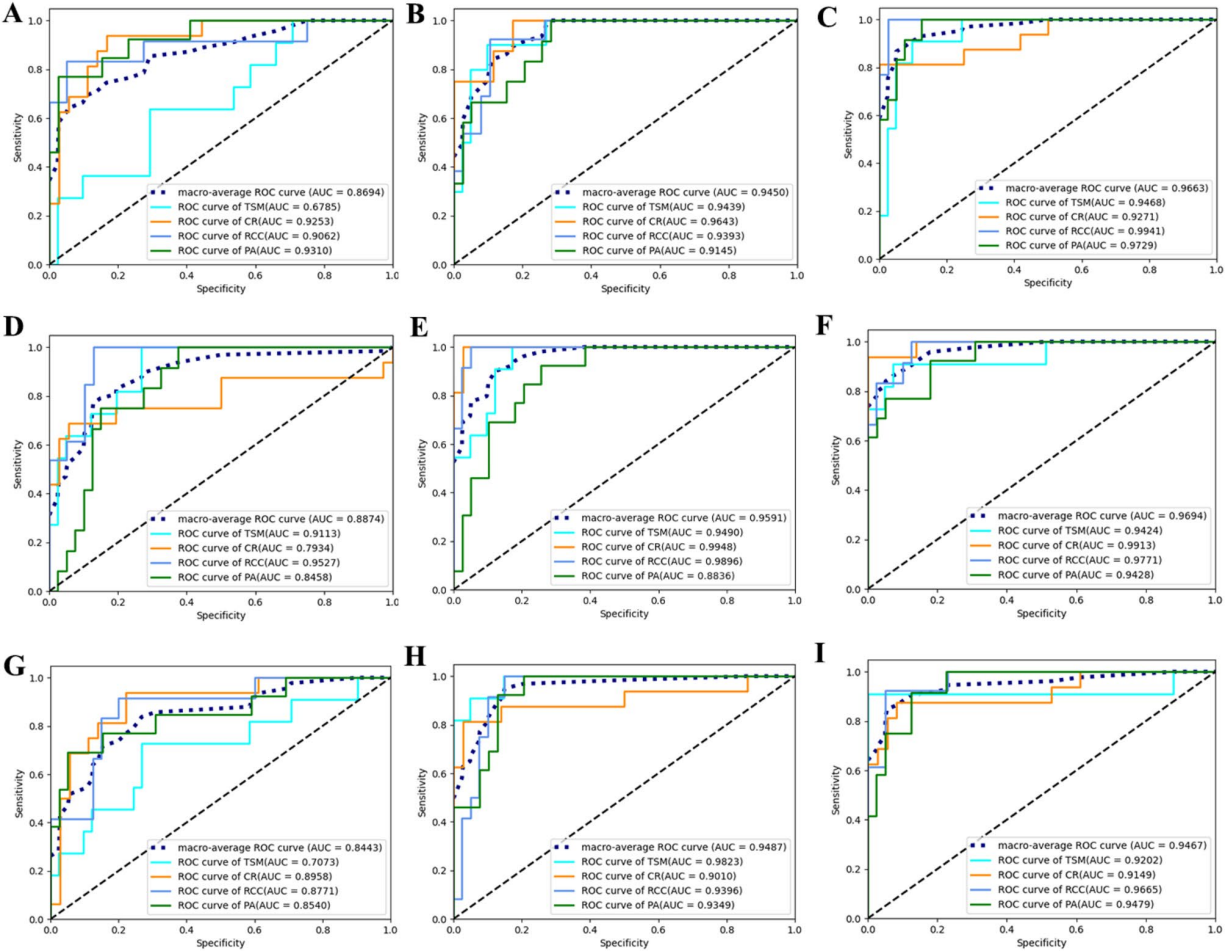
Macroaverage curves of these ML models in different MRI sequences. The performance of XGBoost model on T1-weighted (**D**), T2-weighted (**E**) and contrast-enhanced T1-weighted (**F**) was better than that of SVM model (**A-C**) and LR model (**G-I**) constructed with the corresponding features. The average AUC values of the XGBoost model on T1-weighted, T2-weighted, and contrast-enhanced T1-weighted sequences were 0.8523, 0.9312, and 0.9560, respectively; for the SVM model, the corresponding values were 0.8460, 0.9247, and 0.9381; and for the LR model, the values were 0.8200, 0.9220, and 0.9285, respectively

According to the balanced accuracy, the XGBoost, SVM and LR models were able to differentiate common sellar lesions with the features from each MRI sequence, while XGBoost showed the best performance (Table 3). More specifically, when using CE-T1WI features, the balanced accuracy was 0.83 in the XGBoost model, which was much higher than that in the SVM model (0.77) and LR model (0.75). In the validation set, the mean confusion matrix of each model in each MRI sequence were calculated by five-fold cross-validation (Fig. 2). Based on the results of the mean confusion matrix, the performance of the XGBoost model (Fig. 2B, E, H) was better than that of the SVM (Fig. 2A, D, G) and LR (Fig. 2C, F, I) models with the features from the T1WI, T2WI, and CE-T1WI sequences. The numbers in the figure indicate the number of patients. The performance of each fold for the confusion matrix of each model in each MRI sequence is shown in supplementary file (Figs. 1, 2 and 3).

**Table 3:** Balanced accuracy in differentiating common sellar lesions in MRI sequences

ML model	MRI sequences		
	T1-weighted	T2-weighted	contrast-enhanced T1-weighted
SVM	0.61	0.72	0.77
XGBoost	0.62	0.75	0.83
LR	0.60	0.72	0.75

### Performance of the XGBoost model in differentiating sellar lesions with features from each MRI sequence

The XGBoost model had good performance in differentiating the four common sellar lesions with the features from each MRI sequence. The sensitivity, specificity, and accuracy in differentiating TSMs, CRs, RCCs and PAs are shown in Table 4. The results showed that there were significant differences in sensitivity (*P* = 0.032) and specificity (*P* = 0.045) when using features from CE-T1WI than when using features from the other two MRI sequences. The accuracy was more than 0.90 in contrast-enhanced T1-weighted, which was not significantly different (*P* = 0.215). The XGBoost model has the best performance when using the CE-T1WI features.

**Table 4:** The performance of XGBoost model in differentiating sellar lesions in each MRI sequence

Variables	MRI sequence	TSMs	CRs	RCCs	PAs	*P*-value
Sensitivity	T1-weighted	0.60	0.62	0.64	0.61	0.726
	T2-weighted	0.76	0.84	0.79	0.76	0.059
	contrast-enhanced T1-weighted	0.87	0.82	0.83	0.78	0.032
Specificity	T1-weighted	0.88	0.82	0.89	0.87	0.432
	T2-weighted	0.91	0.90	0.94	0.90	0.051
	contrast-enhanced T1-weighted	0.94	0.98	0.93	0.93	0.045
Accuracy	T1-weighted	0.82	0.76	0.84	0.79	0.353
	T2-weighted	0.88	0.88	0.91	0.87	0.054
	contrast-enhanced T1-weighted	0.93	0.93	0.92	0.90	0.215

### Macro-average ROC curve in differentiating sellar lesions

**Fig. 4:**
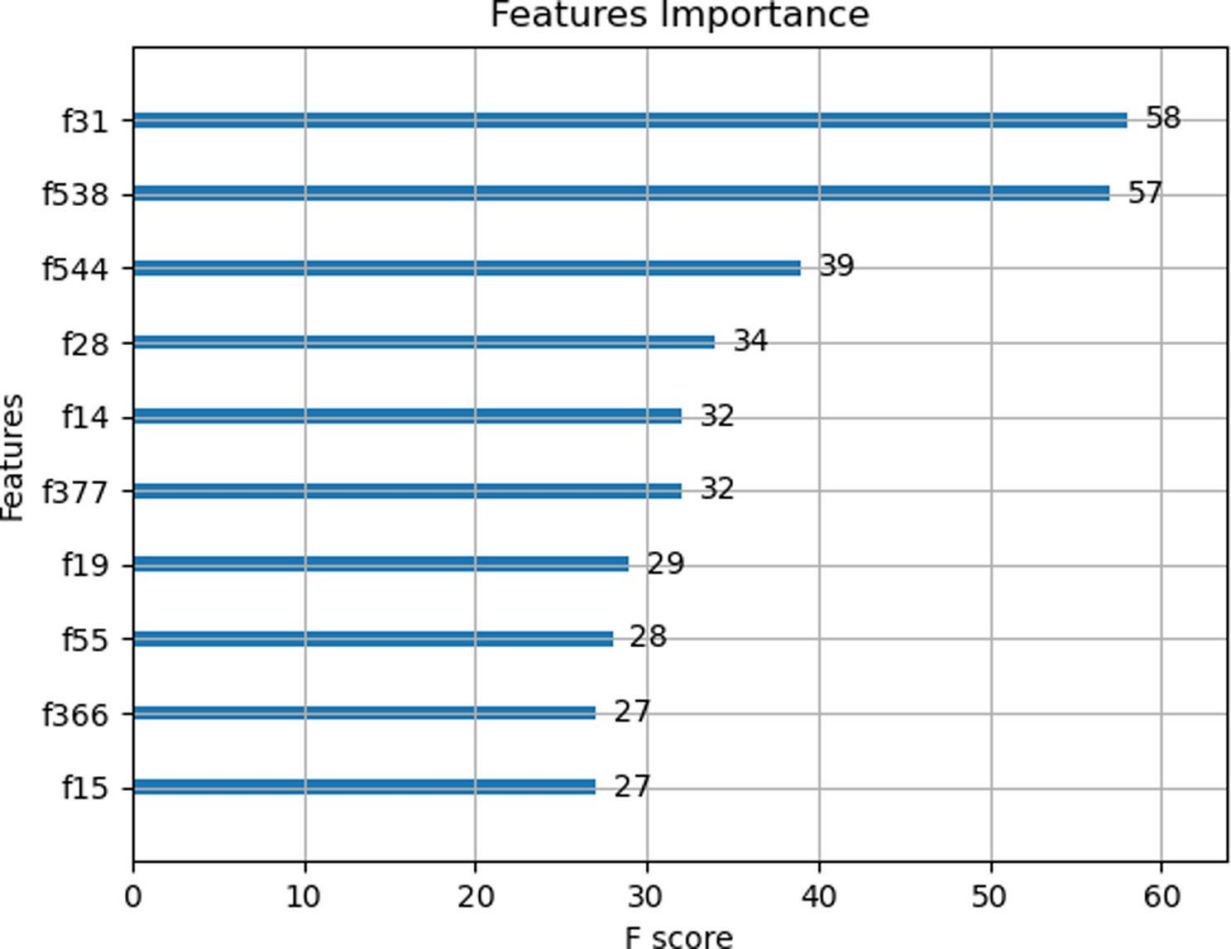
The scores of the top ten important features of the four sellar region lesions. f31: original_firstorder_Variance. f538: wavelet-HHL_firstorder_Mean. f544: wavelet-HHL_firstorder_Skewness. f28: original_firstorder_Skewness. f14: original_firstorder_10Percentile. f377: wavelet-HLL_glcm_JointAverage. f19: original_firstorder_Kurtosis. f55: original_glrlm_GrayLevelNonUniformityNormalized. f366: wavelet-HLL_firstorder_Mean. f15: original_firstorder_90Percentile

**Fig. 5:**
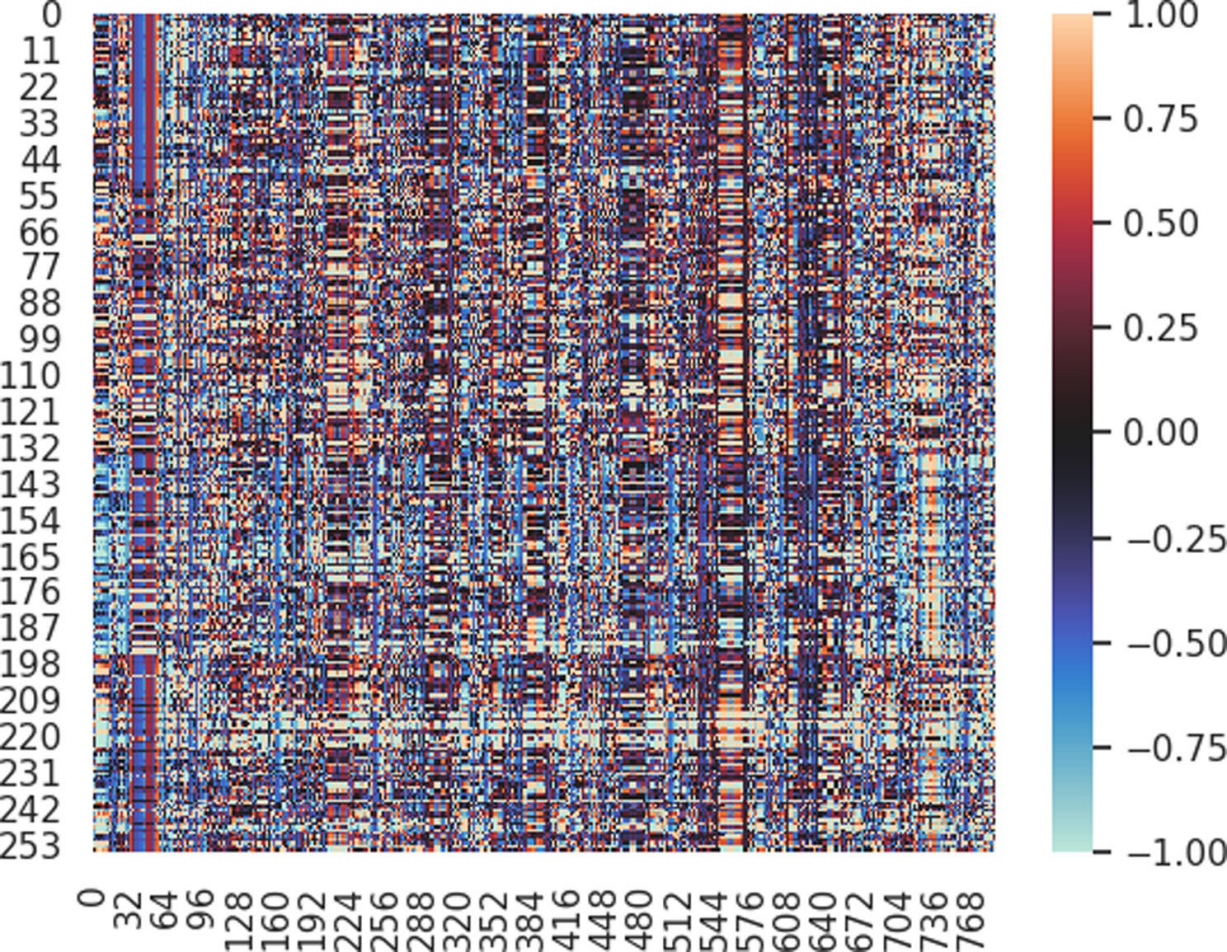
The heatmap of the distribution of each sellar region lesion with contrast-enhanced T1-weighted image radiomics features. Columns represent individual features, while rows correspond to samples. Rows 0–54, 55–135, 136–196, and 197–259 represent patients with TSM, CR, RCC, and PA, respectively

The macro-average shows the performance of the ML models on the entire data set, and it can be used to evaluate the performance of the ML model overall without specifically analyzing the performance in a certain category. The macro-average ROC curves for the training set, derived from different MR sequences and different ML models, all demonstrated AUC values close to 1. Figure 3 shows one of the five-fold cross-validations in the validation set for the three ML models. The XGBoost model’ s macro-average ROC curves for T1-weighted (Fig. 3D), T2-weighted (Fig. 3E), and contrast-enhanced T1-weighted imaging features (Fig. 3F) outperformed those of the SVM (Fig. 3A-C) and LR models (Fig. 3G-I), with all models using corresponding features. The macro-average ROC curve for the evaluation of the other fold for each MR sequence was showed in the supplementary file (Figs. 4, 5, 6). The mean average AUC values of the XGBoost model constructed with T1-, T2-, and contrast-enhanced T1-weighted imaging features were 0.852 (95% CI: 0.816–0.889; Z = 26.92, *P* < 0.001), 0.931 (95% CI: 0.898–0.965; Z = 35.57, *P* < 0.001) and 0.956 (95% CI: 0.942–0.970; Z = 90.57, *P* < 0.001), respectively. In the SVM model, the values were 0.846 (95% CI: 0.827–0.865; Z = 50.68, *P* < 0.001), 0.925 (95% CI: 0.906–0.944; Z = 62.41, *P* < 0.001) and 0.938 (95% CI: 0.915–0.961; Z = 51.70, *P* < 0.001), respectively, while in the LR model, they were 0.820 (95% CI: 0.789–0.851; Z = 28.75, *P* < 0.001), 0.922 (95% CI: 0.884–0.960; Z = 30.92, *P* < 0.001) and 0.929 (95% CI: 0.907–0.950; Z = 55.38, *P* < 0.001) (supplementary file (Table 2)). The macro-average ROC curves showed that the XGBoost model had the best overall performance, followed by the SVM model and the LR model. Among them, the models constructed with the CE-T1WI features had the best performance, followed those constructed with T1WI features and T2WI features. The standard error, z-score, and *P*-value for the AUC in each fold are shown in supplementary file (Tables 3, 4, and 5).

### Feature importance

The importance of the radiomic features extracted from the MR images of each patient was analyzed. In the XGBoost model, features original_firstorder_Variance, wavelet-HHL_firstorder_Mean and wavelet-HHL_firstorder_Skewness were the most important features in the differentiation among the four sellar lesions, with F scores (average gain in all trees, importance score) for 58, 57 and 39, respectively (Fig. 4). The covariance analysis heatmap indicates outstanding performance with CE-T1WI radiomics features, capable of distinguishing TSMs, CRs, RCCs and PAs. Figure 5 shows the sample distribution. Different colors represent different values, which were used to determine the approximate distribution of each sellar region lesion.

### Inference time analysis

The average inference time per case was approximately 0.1 s.

## Discussion

This study investigated the potential value of radiomics-based analysis in differentiating sellar region lesions by using various ML models. We found that the performance of the XGBoost model was superior to that of the SVM and LR models in distinguishing TSMs, CRs, RCCs and PAs. Furthermore, CE-T1WI features appeared to be more useful in differentiating sellar region lesions than T1WI and T2WI features.

MRI is often used as a diagnostic modality in evaluating sellar region lesions. Contrast-enhanced MRI may help neurosurgeons understand the radiographic characteristics of PAs, CRs, RCCs and TSMs [18–20]. However, common diseases in the sellar region may be difficult to distinguish due to the similar imaging features on conventional MRI (both with and without contrast) [5]. Due to the characteristics of cystic contents, the signals of RCCs on MRI are different. In addition, it is difficult to differentiate RCCs and CRs when the walls of the RCC are enhanced and surrounded by the enhanced normal pituitary gland on contrast-enhanced MRI [21]. Additionally, cystic PAs may show different intensities on T1- or T2-weighted images, especially hemorrhagic changes, which is similar to the findings for RCCs and CRs on MR images. ML could provide better performance in classifying and predicting PA subtypes on T2-weighted sequences [6, 7]. Ma et al. [22] used a ML algorithm to achieve good performance in predicting the CR subtype on contrast-enhanced T1-weighted sequences. Huang et al. [4] showed good performance in diagnosing pathological subtypes of CR on T1-weighted images. However, there has been only one study [8] on the use of ML for anterior skull base lesions based on contrast-enhanced T1-weighted sequences. In our study, the ML model was used to differentiate PAs and CRs, TSMs and CRs, and PAs and RCCs separately, with accuracies of 0.8, 0.819 and 0.836, respectively. Therefore, the results were only for comparisons between two kinds of anterior skull base lesions, and the performance in differentiating all four anterior skull base lesions from each other was not truly determined. In our study, three models were established to verify their performance in differentiating the four sellar region lesions. The results showed that the XGBoost model had the best performance, and its balanced accuracy with contrast-enhanced T1-weighted sequence features was up to 83%. The average AUC value was 0.9560, which is better than that in the above literature.

The XGBoost framework, proposed by Chen et al. [23], is an efficient and scalable tree-enhanced ML system that is provided in the form of an open-source software package. Its features include distributed processing and high prediction accuracy; it can be adapted to high-dimensional features and can effectively prevent overfitting. The impact of this system has been widely recognized in many fields, especially environmental analysis [24], molecular biology [25, 26], and neuroimaging [10, 27]. The benefit of using the XGBoost model is that an importance score for each attribute can be calculated, which represents the value of the related attribute in building the enhanced decision tree within the model. In this study, by calculating the importance scores of the extracted imaging features, especially for adjusting the parameters of XGBoost, sparse large-scale image feature data are processed efficiently, and the flexibility of distributed and parallel computing is realized. The optimal XGBoost identification model is obtained by using a series of decision trees to estimate the target features and define quantized weights for each leaf node. However, the lack of a formal ablation study remains a limitation, as it limits detailed insights into the contribution of individual feature subsets. In future studies—especially when extending feature extraction across imaging sequences or modalities—integrating cross-domain attention-guided fusion and optimization strategies may offer more interpretable and efficient feature selection mechanisms [28].

Buchlak et al. [11] demonstrated that the most commonly used ML algorithms in neurosurgery currently include LR, SVM and neural networks. However, the accuracy of the prediction from LR was 76.17 (14.32; 12), and that of SVM was 81.85 (6.72; 18); while SVM was superior to LR, both can achieve good performance in classification tasks. However, although SVM is flexible in handling complex feature relationships, it is prone to overfitting. In this study, the balanced accuracy was 0.75 for LR and 0.77 for SVM, consistent with the values observed in a previous study [11]. The main advantage of the SVM model is that it can model medium, nonlinear relationships, while LR is limited to linear relationships. It has been noted that the LR model is usually the preferred algorithm for predicting the results of binary classification tasks [11]. This study focused on the simultaneous differentiation of four sellar region diseases, and the results showed that the XGBoost model had the best performance, followed by the SVM model and the LR model. The reasons are two-fold: (1) XGBoost adds regular terms into the objective function to control the complexity of the model and avoid overfitting; (2) XGBoost supports column sampling, that is, random selection of features, which enhances model stability.

Many studies [29, 30] have established ML models for PAs and CRs, but only with one kind of MRI sequence. Machad et al. [31] showed that the accuracy in predicting the postoperative recurrence of nonfunctional PAs was 96.3% when using contrast-enhanced T1-weighted MRI. Another study [29] achieved AUC values ranging from 0.608 to 0.781 with seven ML models in predicting recurrence in Cushing’s syndrome patients after sphenoidal based on contrast-enhanced T1-weighted MRI. Due to the different signal characteristics of these sellar region lesions, contrast-enhanced T1-weighted MRI is the best sequence for the image-based identification of these diseases. In this study, contrast-enhanced T1-weighted MRI was also the best MRI sequence among conventional MRI sequences, which is consistent with the above studies. The reason may be that contrast-enhanced T1-weighted MRI can highlight the outlines of the lesions, which could be analyzed by ML models using shape and texture features. However, Kitajima et al. [9] reported that the AUC was 0.990 with their artificial neural network in differentiating PAs, CRs and RCCs with contrast-enhanced T1-weighted imaging features. In our study, the mean average AUC with the XGBoost model built from contrast-enhanced T1-weighted sequence features was 0.9560, much lower than the value above. In that study, however, only three sellar region lesions were included, in contrast to the four we differentiated with our ML models, which may explain the performance differences between the studies.

Our study has several limitations that warrant consideration. First, although the dataset of sellar region lesions was relatively large and comprised four pathological subtypes, the number of cases within each subtype was limited, which may increase the risk of overfitting. Second, the data distribution was imbalanced across classes. While we applied the SMOTE algorithm to mitigate this issue, synthetic data may not fully replicate the distribution of real-world clinical samples. Third, all imaging data were obtained from a single center using a specific MRI scanner, which may introduce site-specific biases and limit the generalizability of our findings. Additionally, the absence of external validation restricts our ability to assess the model’s robustness across heterogeneous populations and imaging protocols.

From a technical perspective, although the proposed model achieved high diagnostic performance and a fast inference time of 0.1 s per case, its integration into clinical workflows remains challenging. The current pipeline requires high-performance GPU hardware, manual ROI delineation, and lacks interoperability with radiology information systems such as PACS. In future work, we plan to (1) conduct external validation using multicenter, multi-vendor MRI datasets to enhance generalizability, (2) incorporate automated or semi-automated segmentation tools to reduce human variability and improve workflow efficiency, and (3) integrate the model into real-time clinical platforms to facilitate seamless deployment. Additionally, integrating generative models to improve image quality and enhance latent representations may benefit automatic segmentation performance, especially in low-contrast regions [32].

## Conclusions

Our study demonstrated that the XGBoost model, using radiomics features from contrast-enhanced T1-weighted MR images, outperformed SVM and LR models in differentiating common sellar region lesions. The proposed model may have potential as a decision-support tool to aid in diagnostic evaluation. However, given the study’ s single-center design, further validation using larger, multi-center datasets is essential to confirm its robustness and clinical applicability.

## Electronic supplementary material

Below is the link to the electronic supplementary material.

Supplementary Material 1

## Data Availability

The datasets used and/or analyzed during the current study are available from the corresponding author upon reasonable request.

## Abbreviations

AUC: Area under the curve
CE-T1WI: Contrast-enhanced T1-weighted images
CRs: Craniopharyngiomas
ICC: Intraclass correlation coefficient
LR: Logistic regression
ML: Machine learning
MRI: Magnetic resonance imaging
PAs: Pituitary adenomas
RCCs: Rathke’s cleft cysts
ROC: Receiver operating characteristic
SVM: Support vector machine
T1WI: T1-weighted images
T2WI: T2-weighted images
TNR: True negative rate
TPR: True positive rate
TSMs: Tuberculum sellar meningiomas
XGBoost: Extreme gradient boosting
