# Supplementary material for: Radiomic study of common sellar region lesions differentiation in magnetic resonance imaging based on multi-classification machine learning model
Source: BMC Med Imaging. 2025 May 3;25:147. doi: 10.1186/s12880-025-01690-5 (PMC12049783; doi:10.1186/s12880-025-01690-5)
Supplement: Supplementary file 1 — Supplementary Material 1 [file 12880_2025_1690_MOESM1_ESM.docx]

**SUPPLEMENTAL ONLINE DATA**

Radiomic study of common sellar region lesions differentiation in magnetic resonance imaging based on multi-classification machine learning model

**This supplementary material includes:**

- **Detailed statistical methodology.**
- **Table 1. Comparison of hormone levels in different sellar region lesions**
- **Table 2. The macro-AUC in differentiating common Sellar lesions in MRI sequences**
- **Table 3. The AUC of each fold of the LR model in differentiating common Sellar lesions in MRI sequences**
- **Table 4. The AUC of each fold of the XGBoost model in differentiating common Sellar lesions in MRI sequences**
- **Table 5. The AUC of each fold of the SVM model in differentiating common Sellar lesions in MRI sequences**
- **Table 6. The comparison of our model with the previous deep learning models**
- **Fig.1 The confusion matrix of SVM model**
- **Fig.2 The confusion matrix of XGBoost model**
- **Fig.3 The confusion matrix of LR model**
- **Fig.4 The macro-average ROC curve of the other fold of the SVM model**
- **Fig.5 The macro-average ROC curve of the other fold of the XGBoost model**
- **Fig.6 The macro-average ROC curve of the other fold of the LR model**
- **Detailed statistical methodology.**

To validate the performance of our classification model, we calculated the area under the receiver operating characteristic (ROC) curve (AUC). We adopted the nonparametric analytical approach by Hanley & McNeil [1], which is robust to distributional assumptions and widely used in radiology and biomedical informatics.

To evaluate whether the AUCs in each fold were significantly better than random guessing (AUC = 0.5), we adopted the z-score–based approach described by Hanley & McNeil [1].

The macro-averaged AUC was further analyzed using a t-distribution–based confidence interval and z-test, assuming approximate normality of fold-level estimates under the central limit theorem (Lasko et al., 2005) [2].

**References:**

[1] Hanley, J. A., & McNeil, B. J. (1982). The meaning and use of the area under a receiver operating characteristic (ROC) curve. Radiology, 143(1), 29–36. <https://doi.org/10.1148/radiology.143.1.7063747>

[2] Lasko, T. A., Bhagwat, J. G., Zou, K. H., & Ohno-Machado, L. (2005). The use of receiver operating characteristic curves in biomedical informatics. Journal of biomedical informatics, 38(5), 404–415. <https://doi.org/10.1016/j.jbi.2005.02.008>

**Table 1.** Comparison of hormone levels in different sellar region lesions

|  | Range of normal values | TSM  (n=54) | CR  (n=81) | RCCs  (n=61) | PAs  (n=63) |
| --- | --- | --- | --- | --- | --- |
| GH(ng/ml) | 0.030-2.47 | 0.80±0.44 | 0.76±0.44 | 0.67±0.37 | 0.70±0.44 |
| Cor(nmol/L) | 133-537 | 264.11±70.82 | 271.72±76.49 | 286.69±69.19 | 275.97±102.01 |
| PRL(ng/ml) | 4.6-21.4 | 17.26±4.185 | 17.13±7.64 | 14.04±4.35 | 16.54±8.22 |
| TTE(ng/ml) | 2.50-9.08 | 4.98±1.94 | 4.53±1.77 | 4.24±1.46 | 4.48±1.37 |
| LH(IU/L) | 1.7-8.6 | 4.25±2.46 | 4.14±1.46 | 3.89±1.26 | 4.26±1.41 |
| FSH(pg/ml) | 11.3-43.2 | 21.80±7.82 | 22.30±3.64 | 22.21±6.09 | 20.34±4.09 |
| TSH (mIU/L) | 0.27-4.2 | 1.24±0.46 | 1.18±0.67 | 1.04±0.65 | 1.12±0.55 |
| T3(mmol/L) | 1.3-3.1 | 1.94±0.89 | 1.80±0.43 | 1.83±0.45 | 1.9±0.46 |
| FT3(pmol/L) | 3.60-7.50 | 4.76±0.95 | 4.83±0.92 | 5.05±0.61 | 4.97±0.82 |
| Thyroxine (nmol/L) | 64-164 | 105.62±34.64 | 116.72±28.75 | 116.21±29.86 | 109.84±28.13 |
| FT4(pmol/L) | 12-22 | 16.41±2.56 | 14.83±0.92 | 15.05±0.61 | 15.00±0.63 |

GH = Growth hormone, Cor = Cortisol, PRL = Prolactin, TTE = Testosterone, LH = Lutein, FSH = Follicle-stimulating hormone, TSH = Thyroid stimulating hormone, T3 = Triiodothyronine, FT3 = Free triiodothyronine, FT4 = Free thyroxine.

**Table 2.** The macro-AUC in differentiating common Sellar lesions in MRI sequences

| ML model | MRI sequences | | | | | | | | |
| --- | --- | --- | --- | --- | --- | --- | --- | --- | --- |
|  | T1-weighted | | | T2-weighted | | | contrast-enhanced T1-weighted | | |
|  | AUC(95%CI) | Z-score | P-value | AUC(95%CI) | Z-score | P-value | AUC(95%CI) | Z-score | P-value |
| SVM | 0.846  (0.827-0.865) | 50.68 | < 0.001 | 0.925  (0.906-0.944) | 62.41 | < 0.001 | 0.938  (0.915-0.961) | 51.7 | < 0.001 |
| XGBoost | 0.852 (0.816-0.889) | 26.92 | < 0.001 | 0.931  (0.898–0.965) | 35.57 | < 0.001 | 0.956  (0.942-0.970) | 90.57 | < 0.001 |
| LR | 0.820  (0.789-0.851) | 28.75 | < 0.001 | 0.922  (0.884-0.960) | 30.92 | < 0.001 | 0.929  (0.907-0.950) | 55.38 | < 0.001 |

**Table 3.** The AUC of each fold of the LR model in differentiating common Sellar lesions in MRI sequences

| Subfold | MRI sequences | | | | | | | | | | | |
| --- | --- | --- | --- | --- | --- | --- | --- | --- | --- | --- | --- | --- |
|  | T1-weighted | | | | T2-weighted | | | | contrast-enhanced T1-weighted | | | |
|  | AUC | SE(AUC) | Z-score | P-value | AUC | SE(AUC) | Z-score | P-value | AUC | SE(AUC) | Z-score | P-value |
| 1 | 0.838 | 0.073 | 4.624 | <0.001 | 0.872 | 0.066 | 5.604 | <0.001 | 0.902 | 0.059 | 6.797 | <0.001 |
| 2 | 0.782 | 0.082 | 3.459 | 0.001 | 0.940 | 0.047 | 9.326 | <0.001 | 0.923 | 0.053 | 7.976 | <0.001 |
| 3 | 0.826 | 0.075 | 4.324 | <0.001 | 0.934 | 0.049 | 8.805 | <0.001 | 0.940 | 0.047 | 9.326 | <0.001 |
| 4 | 0.810 | 0.078 | 3.982 | <0.001 | 0.915 | 0.056 | 7.463 | <0.001 | 0.930 | 0.051 | 8.480 | <0.001 |
| 5 | 0.844 | 0.072 | 4.780 | <0.001 | 0.949 | 0.044 | 10.219 | <0.001 | 0.947 | 0.045 | 9.991 | <0.001 |

**Table 4.** The AUC of each fold of the XGBoost model in differentiating common Sellar lesions in MRI sequences

| Subfold | MRI sequences | | | | | | | | | | | |
| --- | --- | --- | --- | --- | --- | --- | --- | --- | --- | --- | --- | --- |
|  | T1-weighted | | | | T2-weighted | | | | contrast-enhanced T1-weighted | | | |
|  | AUC | SE(AUC) | Z-score | P-value | AUC | SE(AUC) | Z-score | P-value | AUC | SE(AUC) | Z-score | P-value |
| 1 | 0.875 | 0.066 | 5.689 | <0.001 | 0.931 | 0.050 | 8.534 | <0.001 | 0.963 | 0.037 | 12.366 | <0.001 |
| 2 | 0.827 | 0.075 | 4.347 | <0.001 | 0.920 | 0.054 | 7.770 | <0.001 | 0.944 | 0.046 | 9.712 | <0.001 |
| 3 | 0.853 | 0.070 | 5.007 | <0.001 | 0.954 | 0.042 | 10.890 | <0.001 | 0.945 | 0.045 | 9.774 | <0.001 |
| 4 | 0.820 | 0.076 | 4.203 | <0.001 | 0.892 | 0.062 | 6.358 | <0.001 | 0.959 | 0.040 | 11.553 | <0.001 |
| 5 | 0.887 | 0.063 | 6.158 | <0.001 | 0.959 | 0.039 | 11.649 | <0.001 | 0.969 | 0.034 | 13.701 | <0.001 |

**Table 5.** The AUC of each fold of the SVM model in differentiating common Sellar lesions in MRI sequences

| Subfold | MRI sequences | | | | | | | | | | | |
| --- | --- | --- | --- | --- | --- | --- | --- | --- | --- | --- | --- | --- |
|  | T1-weighted | | | | T2-weighted | | | | contrast-enhanced T1-weighted | | | |
|  | AUC | SE(AUC) | Z-score | P-value | AUC | SE(AUC) | Z-score | P-value | AUC | SE(AUC) | Z-score | P-value |
| 1 | 0.843 | 0.072 | 4.754 | <0.001 | 0.934 | 0.049 | 8.780 | <0.001 | 0.932 | 0.050 | 8.580 | <0.001 |
| 2 | 0.834 | 0.074 | 4.518 | <0.001 | 0.909 | 0.057 | 7.159 | <0.001 | 0.946 | 0.045 | 9.946 | <0.001 |
| 3 | 0.832 | 0.074 | 4.468 | <0.001 | 0.925 | 0.053 | 8.070 | <0.001 | 0.930 | 0.051 | 8.473 | <0.001 |
| 4 | 0.851 | 0.071 | 4.971 | <0.001 | 0.911 | 0.057 | 7.245 | <0.001 | 0.917 | 0.055 | 7.562 | <0.001 |
| 5 | 0.869 | 0.067 | 5.511 | <0.001 | 0.945 | 0.045 | 9.806 | <0.001 | 0.966 | 0.036 | 12.989 | <0.001 |

**Table 6.** The comparison of our model with the previous deep learning models

| Study | AI model used | Accuracy/Balanced accuracy (%) | AUC/macro-AUC | Dataset size | Classification tasks |
| --- | --- | --- | --- | --- | --- |
| Zhu et al., 2020 [3] | Semi supervised model (CycleGAN) | 73.8 | 0.71 | 55 | the softness level of pituitary tumors |
| Jiang et al., 2020 [4] | Radiomics & deep learning hybrid model | 79.5 | 0.75 | 399 | Pituitary apoplexy, cystic pituitary adenoma (cysticA), Rathke’s cleft cyst, and cystic craniopharyngioma |
| Our model | SVM, XGBoost, LR | 83.0 | 0.956 | 259 | Pituitary adenoma, craniopharyngioma, Rathke’s cleft cysts, and tuberculum sellar meningiomas |

**References:**

[3] Zhu, H., Fang, Q., Huang, Y., & Xu, K. (2020). Semi-supervised method for image texture classification of pituitary tumors via CycleGAN and optimized feature extraction. BMC medical informatics and decision making, 20(1), 215. https://doi.org/10.1186/s12911-020-01230-x

[4] Jiang, C., Zhang, W., Wang, H., Jiao, Y., Fang, Y., Feng, F., Feng, M., & Wang, R. (2023). Machine Learning Approaches to Differentiate Sellar-Suprasellar Cystic Lesions on Magnetic Resonance Imaging. Bioengineering (Basel, Switzerland), 10(11), 1295. https://doi.org/10.3390/bioengineering10111295


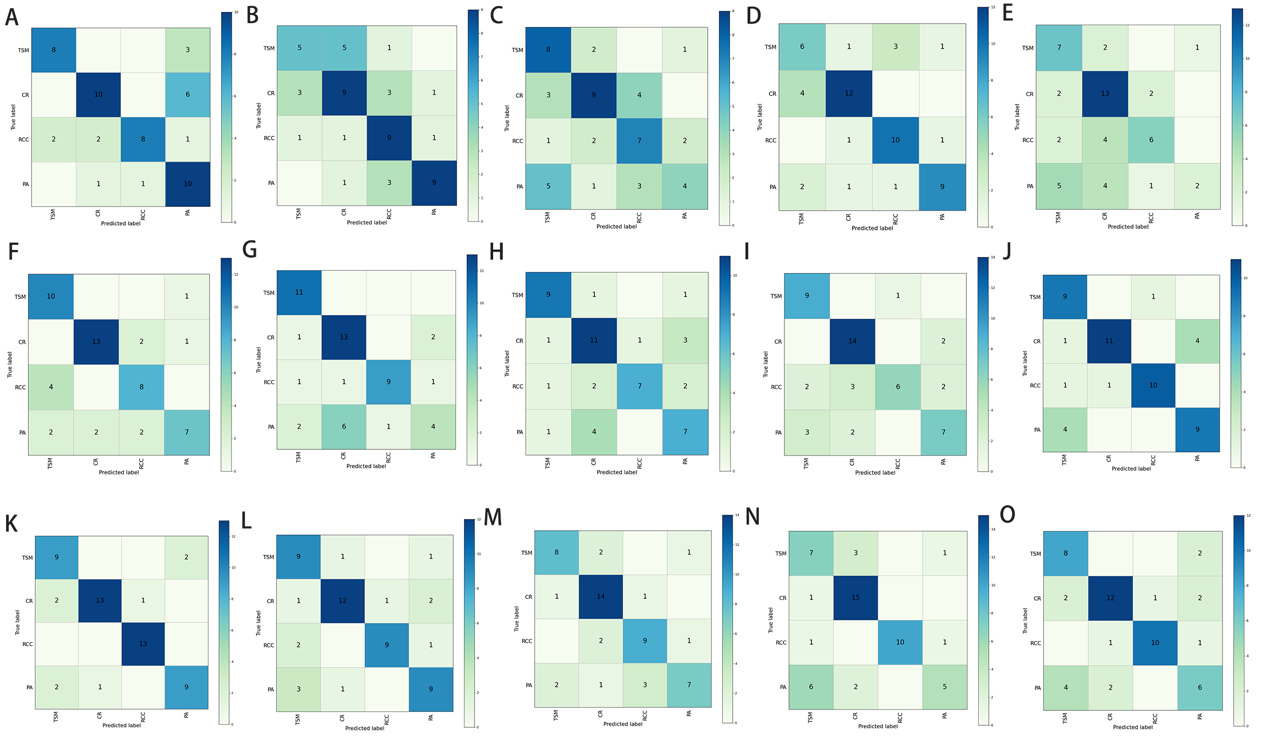


**Fig.1** The confusion matrix of SVM model was calculated by five-fold cross-validation with T1-weighted imaging (Fig.1A-E), T2-weighted imaging (Fig.1F-J) and contrast-enhanced T1-weighted imaging features (Fig.1K-O).


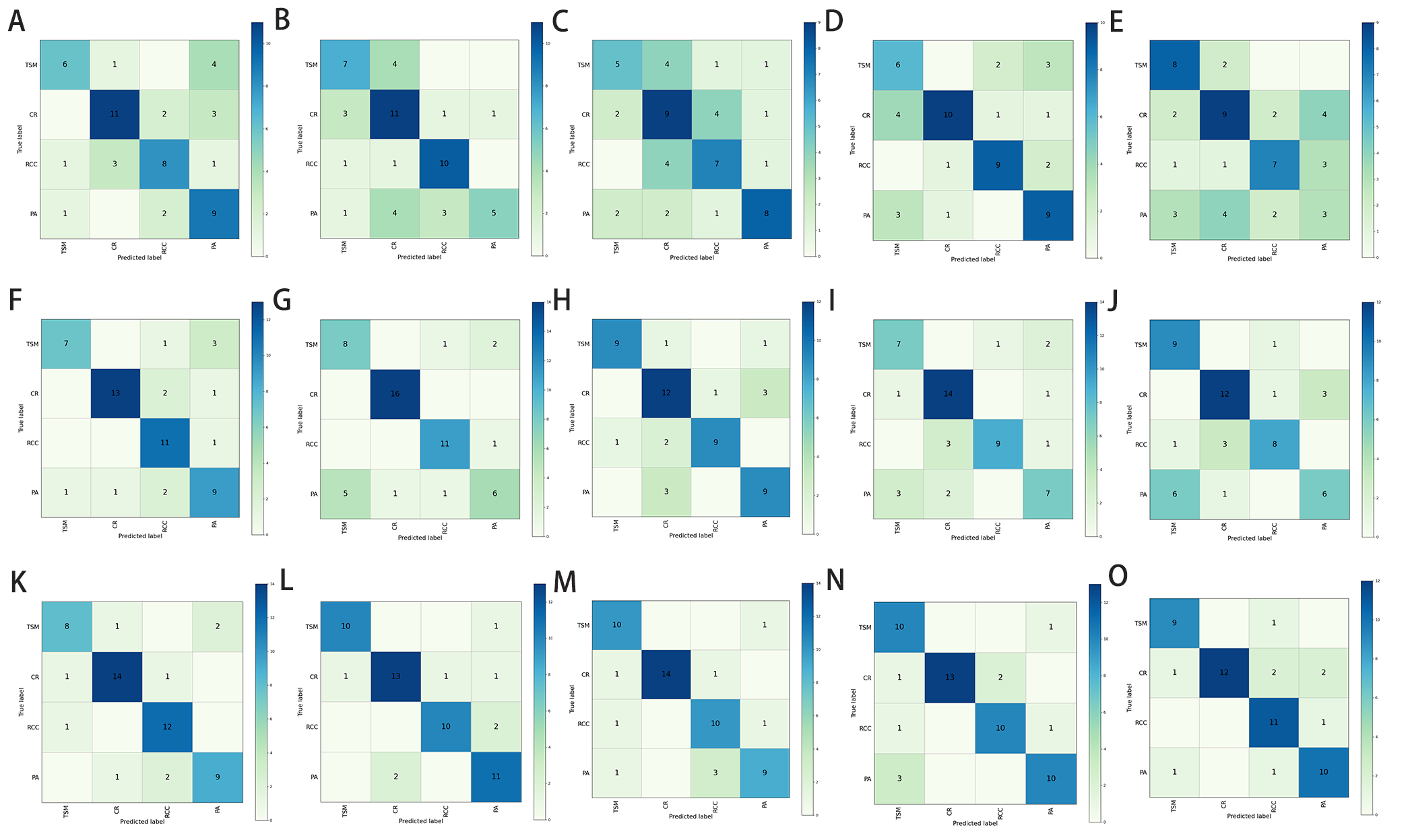


**Fig.2** The confusion matrix of XGBoost model was calculated by five-fold cross-validation with T1-weighted imaging (Fig.2A-E), T2-weighted imaging (Fig.2F-J) and contrast-enhanced T1-weighted imaging features (Fig.2K-O).


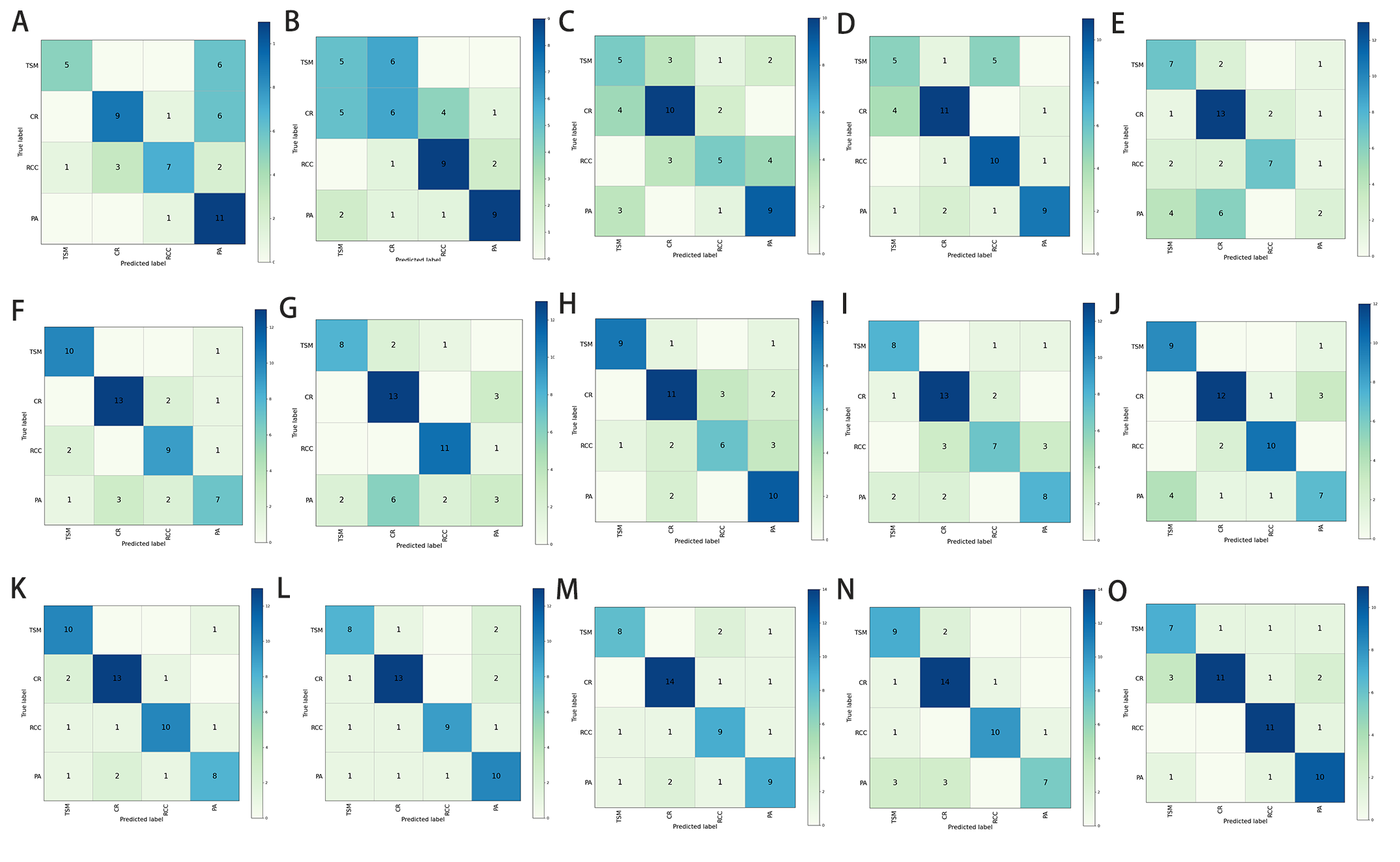


**Fig.3** The confusion matrix of LR model was calculated by five-fold cross-validation with T1-weighted imaging (Fig.3A-E), T2-weighted imaging (Fig.3F-J) and contrast-enhanced T1-weighted imaging features (Fig.3K-O).


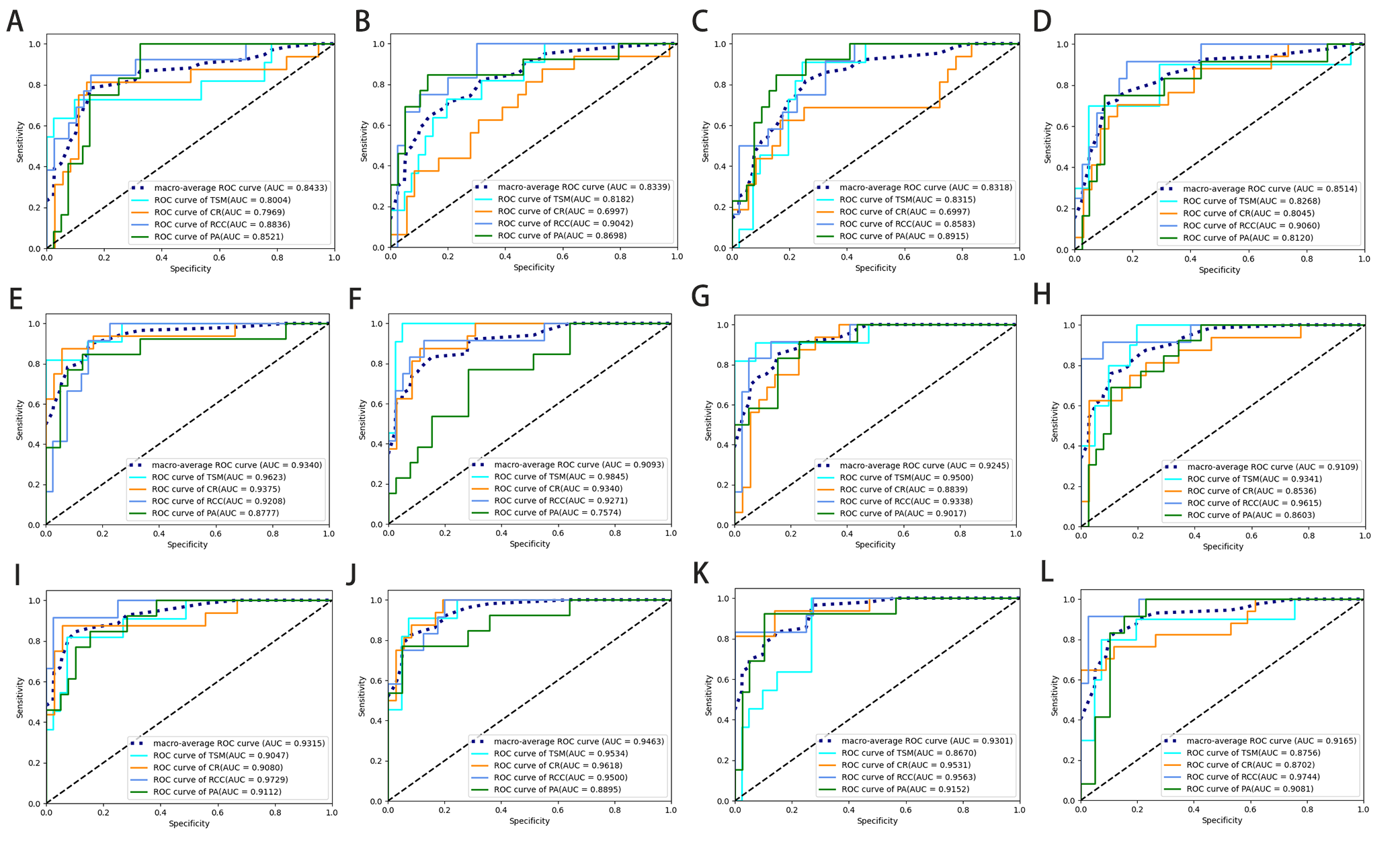


**Fig.4** The macro-average ROC curve of the other fold of the SVM model with T1-weighted imaging (Fig.4A-D), T2-weighted imaging (Fig.4E-H) and contrast-enhanced T1-weighted imaging features (Fig.4I-L).


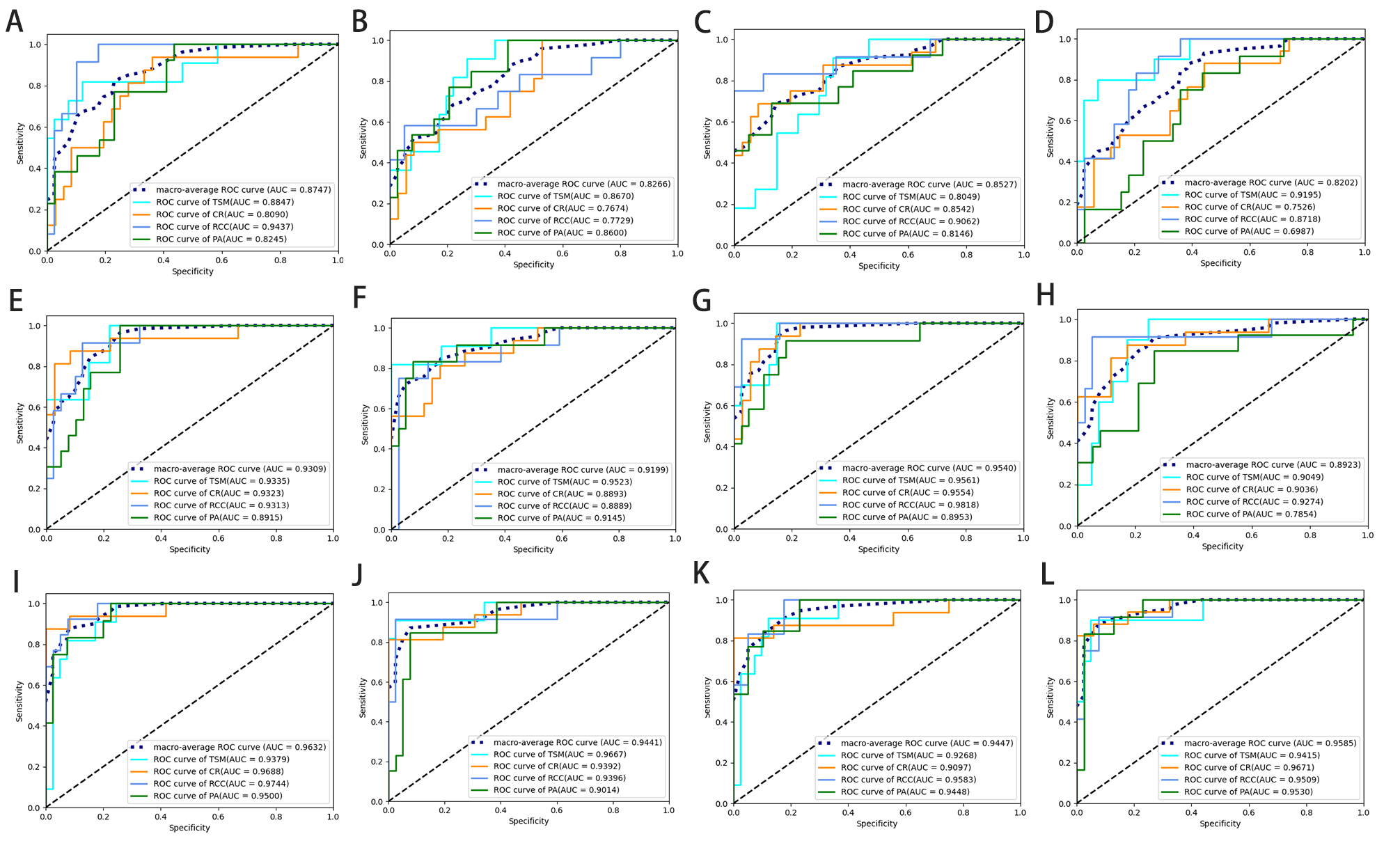


**Fig.5** The macro-average ROC curve of the other fold of the XGBoost model with T1-weighted imaging (Fig.5A-D), T2-weighted imaging (Fig.5E-H) and contrast-enhanced T1-weighted imaging features (Fig.5I-L).


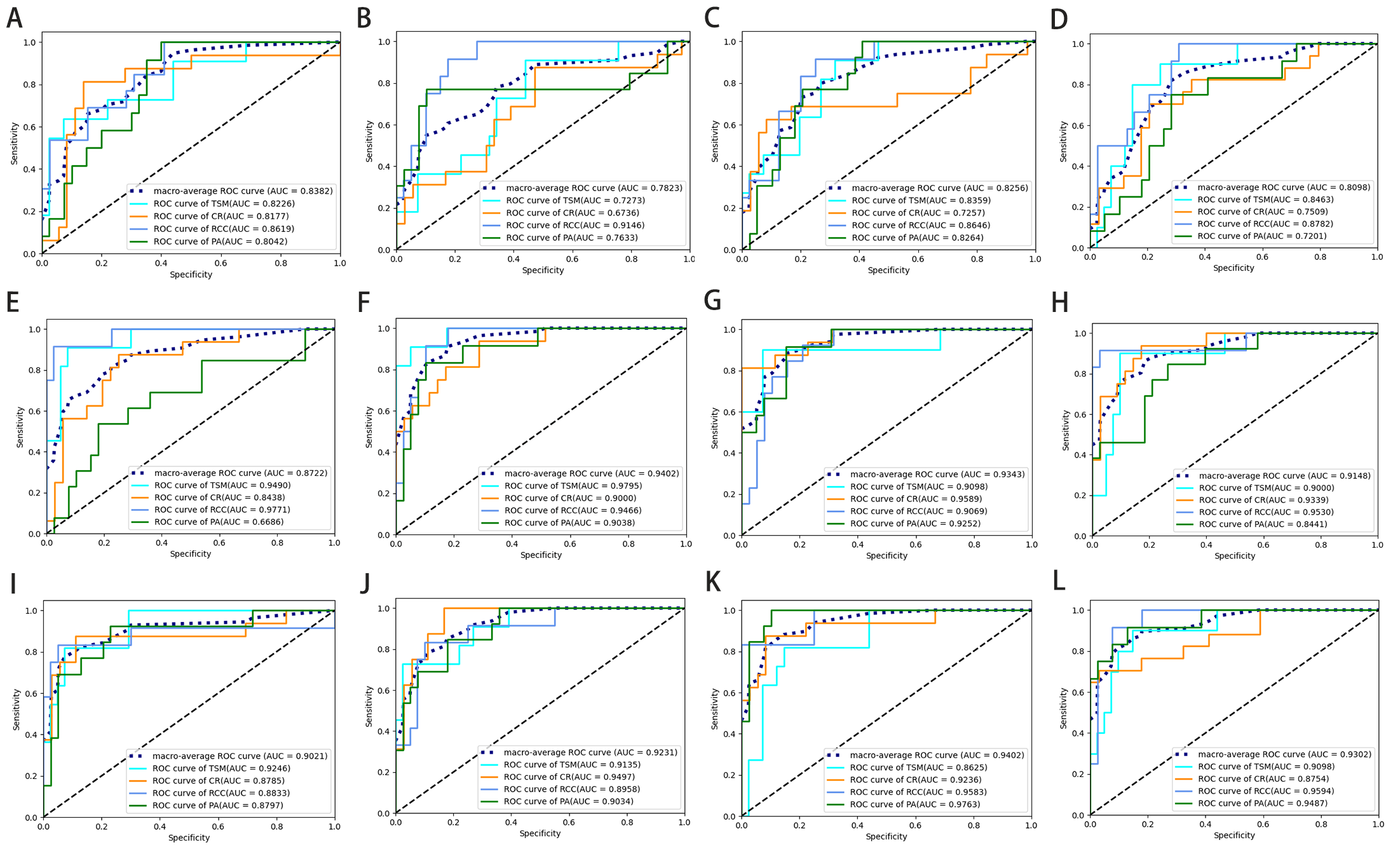


**Fig.6** The macro-average ROC curve of the other fold of the LR model with T1-weighted imaging (Fig.6A-D), T2-weighted imaging (Fig.6E-H) and contrast-enhanced T1-weighted imaging features (Fig.6I-L).
